# Deficiency of telomere-associated repressor activator protein 1 precipitates cardiac aging in mice *via* p53/PPARα signaling

**DOI:** 10.7150/thno.51739

**Published:** 2021-03-04

**Authors:** Yin Cai, Hao Liu, Erfei Song, Lin Wang, Jindong Xu, Yi He, Dengwen Zhang, Liyan Zhang, Kenneth King-yip Cheng, Leigang Jin, Min Wu, Shiming Liu, Dake Qi, Liangqing Zhang, Gary D. Lopaschuk, Sheng Wang, Aimin Xu, Zhengyuan Xia

**Affiliations:** 1 State Key Laboratory of Pharmaceutical Biotechnology, LKS Faculty of Medicine, The University of Hong Kong, Hong Kong SAR, China.; 2 Department of Anaesthesiology, LKS Faculty of Medicine, The University of Hong Kong, Hong Kong SAR, China.; 3 Department of Health Technology and Informatics, The Hong Kong Polytechnic University, Hong Kong SAR, China.; 4 Department of Anaesthesiology, The Second Affiliated Hospital of Guangzhou Medical University, Guangzhou Institute of Cardiovascular Disease, Guangzhou, Guangdong, China.; 5 The First Affiliated Hospital, Jinan University, Guangzhou, Guangdong, China.; 6 Department of Anesthesiology, Guangdong Cardiovascular Institute, Guangdong Provincial People's Hospital, Guangdong Academy of Medical Sciences, Guangdong, China.; 7 Cardiovascular Research Centre, University of Alberta, Edmonton, Alberta, Canada.; 8 Department of Medicine, LKS Faculty of Medicine, The University of Hong Kong, Hong Kong SAR, China.; 9 Department of Cardiac Surgery, Guangdong Cardiovascular Institute, Guangdong Provincial People's Hospital, Guangdong Academy of Medical Sciences, Guangdong, China.; 10 Department of Cardiology, The Second Affiliated Hospital of Guangzhou Medical University, Guangzhou Institute of Cardiovascular Disease, Guangzhou, Guangdong, China.; 11 Division of Biomedical Sciences, Faculty of Medicine, Memorial University of Newfoundland, Newfoundland, Canada.; 12 Department of Anesthesiology, Affiliated Hospital of Guangdong Medical University, Zhanjiang, China.; 13 Shenzhen Institute of Research and Innovation, The University of Hong Kong, Shenzhen, China.

**Keywords:** Rap1, p53, PPARα, fatty acid metabolism, cardiac aging

## Abstract

**Background:** Telomere shortening and dysfunction may cause metabolic disorders, tissue damage and age-dependent pathologies. However, little is known about the association of telomere-associated protein Rap1 with mitochondrial energy metabolism and cardiac aging.

**Methods:** Echocardiography was performed to detect cardiac structure and function in Rap1^+/+^ and Rap1^-/-^ mice at different ages (3 months, 12 months and 20 months). Telomere length, DNA damage, cardiac senescence and cardiomyocyte size were analyzed using the real-time PCR, Western blotting, senescence associated β-galactosidase assay and wheat germ agglutinin staining, respectively. Western blotting was also used to determine the level of cardiac fatty acid metabolism related key enzymes in mouse and human myocardium. Chromatin immunoprecipitation assay was used to verify the direct link between p53 and PPARα. The p53 inhibitor, Pifithrin-α and PPARα activator WY14643 were utilized to identify the effects of Rap1/p53/PPARα signaling pathway.

**Results:** Telomere was shortened concomitant with extensive DNA damage in aged Rap1^-/-^ mouse hearts, evidenced by reduced T/S ratios and increased nuclear γH2AX. Meanwhile, the aging-associated phenotypes were pronounced as reflected by altered mitochondrial ultrastructure, enhanced senescence, cardiac hypertrophy and dysfunction. Mechanistically, acetylated p53 and nuclear p53 was enhanced in the Rap1^-/-^ mouse hearts, concomitant with reduced PPARα. Importantly, p53 directly binds to the promoter of PPARα in mouse hearts and suppresses the transcription of PPARα. In addition, aged Rap1^-/-^ mice exhibited reduced cardiac fatty acid metabolism. Pifithrin-α alleviated cardiac aging and enhanced fatty acid metabolism in the aged Rap1^-/-^ mice. Activating PPARα with WY14643 in primarily cultured Rap1^-/-^ cardiomyocytes restored maximal oxygen consumption rates. Reduced Rap1 expression and impaired p53/PPARα signaling also presented in aged human myocardium.

**Conclusion:** In summary, Rap1 may link telomere biology to fatty acid metabolism and aging-related cardiac pathologies *via* modulating the p53/PPARα signaling pathway, which could represent a therapeutic target in preventing/attenuating cardiac aging.

## Introduction

The lifespan of humans is increasing, and the world is experiencing a dramatic increase in the elderly population (people 65 years and older). Meanwhile, the prevalence of cardiovascular diseases increases with aging, which is a leading cause of death in the elderly 1,2. A high density of mitochondria in the heart is required to meet the extraordinary energy demand for adenosine triphosphate (ATP), which is mainly generated by carbohydrate and fatty acids *via* mitochondrial oxidative phosphorylation under physiological conditions 3,4. With aging, the heart exhibits impaired metabolic flexibility and excessive production of reactive oxygen species (ROS), which is associated with reduced fatty acid oxidation and ATP generation 5. As a result, a decrease in the number of cardiomyocytes and the development of left ventricular (LV) hypertrophy/dysfunction occur at the cellular and organ level 6,7. Thus, understanding the mechanisms associated with the impaired fatty acid metabolism in the aging heart may promote the translational process of basic research into clinical practice.

Telomeric repressor activator protein 1 (Rap1), one of the components of the shelterin complex, is associated with telomeres solely through its nuclear interaction with telomeric repeat-binding factor 2 8 for the telomere end protection by repressing aberrant homology-directed repair 9. Rap1 is well known for its pleiotropic functions in apoptosis 10, metabolism 11, inflammation 12,13 and oxidative stress 14,15. However, the role of Rap1, the most conserved telomere-interacting protein, in controlling telomere length remains elusive. Despite reports of comparable telomere length in liver and brown fat in Rap1 deficency mice relative to wildtype mice (30 weeks of age) 11, telomere shortening and the downstream effector p53 (senescence marker) expression is more pronounced in the intestine and perferial blood of both Rap1 and telomerase double knockout mice than in mice with telomerase single mutants (22-30 weeks of age) 16. This implicates Rap1 as playing a crucial role in regulating telomere length in the context of telomerase deficiency. Of note, short telomeres and defective telomerase activity have been involved in the pathobiology of aging-related cardiomyopathy 17,18.

In addition to being a tumour suppressor, p53 deficiency in mouse heart enhances fatty acid metabolism in parallel with an upregulation of peroxisome proliferator-activated receptor α (PPARα) and its co-activator peroxisome proliferator-activated receptor gamma coactivator 1 α (PGC1α) 19. The PPARα/PGC1α complex controls the expression of key enzymes involved in cardiac fatty acid uptake and oxidation 20. Previous study reported that the expressions of PPARα and PGC1α in Rap1-deficient mice were reduced at both the mRNA and protein levels by approximately 50% in the liver and white fat compared to the controls 11. However, the questions as to how deletion of Rap1 is associated with down-regulation of PPARα and PGC1α and whether there is an association between Rap1, p53 and fatty acid metabolism in the aging heart remains unclear. Given that aging is associated with defective telomerease activity, we hypothesized that Rap1 deletion in aged mice may shorten the telomere, thereby increasing functional expression of p53 to accelerate cardiac aging. To address this, we investigated whether or not Rap1 deficiency in mice could impair cardiac fatty acid metabolism and exacerbate cardiac aging. We integrated *in vivo* and *in vitro* approaches with gene knockout and pharmacological interventions. To understand the clinical relevance, we also collected human heart biopsies from young and aged patients undergoing open heart surgeries for mitral valve replacement, heart transplantation or donation, and compared the expression of Rap1, p53, PPARα and fatty acid metabolism-related enzymes.

## Methods and Materials

### Rap1 wildtype and knockout mice and treatment

Rap1 wildtype (Rap1^+/+^) and knockout (Rap1^-/-^) mice were originally donated by Dr. Tergaonkar (Institute of Molecular and Cell Biology, The Agency for Science, Technology and Research, Singapore) 12,13. Genotyping of mouse offspring was performed by polymerase chain reaction (PCR) using genomic DNA extracted from ear biopsies. A 373 bp DNA fragment indicated a homozygous wildtype mouse, while a 229 bp DNA fragment was indicative of the homozygous knockout genotype. Male Rap1^+/+^ and Rap1^-/-^ mice were used in this study. The mice were maintained in C57BL/6N genetic background and reared on a 12 h/12 h light/dark cycle with standard chow (Lab Diet, PMI Nutrition International, Brentwood, MO, USA) and water available *ad libitum* in the Laboratory Animal Unit of The University of Hong Kong. Subgroups of male Rap1^+/+^ and Rap1^-/-^ mice (12 months old) were randomly assigned to treatments with Pifithrin-α (PFTα, a selective inhibitor of p53, Santa Cruz Biotechnology, Dallas, TX, USA) or with dimethyl sulfoxide (DMSO) as the vehicle control group. The mice were administrated PFTα (1.1 mg/kg/day) or DMSO intraperitoneally for 6 weeks. Following this, the mice were subjected to echocardiography. Thereafter, the mouse tissues were harvest after the mice were subjected to deep anesthesia with ketamine (100 mg/kg) & xylazine (10 mg/kg). Blood samples were collected by cardiac puncture with heparinized syringes and centrifuged at 3,000 rpm for 10 min at room temperature to collect the plasma, which was then immediately stored at -80 °C for later analysis. Heart was quickly isolated, snap frozen in liquid nitrogen and stored at -80 °C for further analysis. A separate cohort of male Rap1^+/+^ (n = 24) and Rap1^-/-^ (n = 40) mice were placed on a standard chow diet for an extended time period in order to monitor their lifespan. All experimental procedures were approved by The University of Hong Kong Committee on the Use of Live Animals for Teaching and Research.

### Novel object recognition test

The aged Rap1^+/+^ and Rap1^-/-^ mice (male, 20 months old) were habituated in the open field arena in the absence of objects for 20 min/day for 3 days before being subjected to the novel object recognition test. In the acquisition phase, two identical sample objects (A+A) were placed into the arena at the fixed location and the mice were allowed 10 min to explore the objects. After a retention interval of 24 h, the mice were returned to the same arena with two objects, with one is identical to the sample and the other object was replaced with a new one (A+B). The two objects were of the same size and placed at the same location as the previous ones. The mice were allowed to explore the objects for 10 min. The time spent on each object was recorded and evaluated by an experimenter who was blind to experimental conditions. Recognition memory was evaluated by the ratio of the exploration time between the new and old objects 21.

### Echocardiography

Transthoracic echocardiography was performed noninvasively using a Vevo 2100 high-resolution imaging system equipped with a 30-MHz probe (MS550D; VisualSonics, Toronto, Ontario, Canada), as previously described 10, 22. The Rap1^+/+^ and Rap1^-/-^ mice (male, at different ages: i.e., 3, 12 and 20 months old) were anesthetized with 0.8% isoflurane and maintained on a heating pad with electrocardiogram recording. M-mode echocardiograms were obtained from parasternal short-axis view at the papillary level for measurements of LV end-diastolic diameter (LVID-d) and LV mass. LV ejection fraction (EF) was calculated to evaluate cardiac systolic function. The apical four chamber view was obtained for the assessment of myocardial performance index (MPI), isovolumic relaxation time (IVRT) and ratio of the early (E) to late (A) ventricular filling velocities (E/A ratio). All parameters were averaged over 5 cardiac cycles for analysis.

### Wheat germ agglutinin staining

To analyse cardiomyocyte size, the LV adjacent sections from Rap1^+/+^ and Rap1^-/-^ mice (male, 20 months old) were stained with AlexaFluor-594 conjugated wheat germ agglutinin (WGA, Invitrogen, Carlsbad, CA, USA) 23. Briefly, myocardial slides were de-waxed, rehydrated, and boiled in sodium citrate buffer (10 mM) for 10 min before blocking with 1% (w/v) BSA in phosphate-buffered saline (PBS) for 1 h. The sections were then incubated in WGA (10 µg/mL in PBS) for 1 h in the dark at room temperature. Images of the slides, from five random separate fields, were taken using a BX41 microscope equipped with a DP72 colour digital camera (Olympus, Tokyo, Japan). The average cardiomyocyte size was quantified by measuring cardiomyocyte area (in µm^2^) from those photos with Image J software (National Institutes of Health, Bethesda, MD, USA).

### Senescence associated β-galactosidase assay

Senescence associated β-galactosidase (SA-β-Gal) is a widely used marker for detecting cell senescence 24. The SA-β-Gal staining on frozen sections (5 μm) of the heart from Rap1^+/+^ and Rap1^-/-^ mice (male, 20 months old) was performed with the Senescence β-Galactosidase Staining Kit (Cell Signaling Technology, Danvers, MA, USA) according to the manufacturer's instruction.

### Dihydroethidium staining for superoxide anion production

*In situ* (O_2_^·-^) production was detected by fluorescent probe dihydroethidium (DHE, Sigma-Aldrich, St. Louis, MO, USA) staining as described 25. Briefly, the frozen sections of LV tissue (5 μm) from Rap1^+/+^ and Rap1^-/-^ mice (male, 12 months old) were incubated with DHE (10 μM) at 37 ºC for 30 min. Fluorescence images were obtained using a BX41 microscope equipped with a DP72 colour digital camera (Olympus). The fluorescence intensity of DHE-labelled positive staining was quantified with Image J software in each of five randomly selected fields.

### Immunofluorescence

The frozen LV adjacent sections (5 μm) from Rap1^+/+^ and Rap1^-/-^ mice (male, 12 months old) were fixed with 4% paraformaldehyde and then incubated with 0.1% Triton X-100 for nuclear permeabilization. The tissue sections were then blocked with 5% Goat serum in PBS, incubated sequentially with primary antibodies [p53 (1:2000, Cell Signaling Technology); α-actinin (1:500, Cell Signaling Technology)], secondary antibodies [Goat anti-Mouse Alexa Fluor 594 (1:500, Invitrogen); Goat anti-Rabbit Alexa Fluor 488 (1:500, Invitrogen)] and ProLong^®^ Gold Antifade Mountant with DAPI (Invitrogen). The images were acquired under Leica TCS SPE Confocal Microscope (University Research Facility in Life Sciences, Hong Kong Polytechnic University). Image analysis was performed with Image J software.

### H9C2 cells and isolation of primary cardiomyocytes

H9C2 cells were obtained from the American Type Culture Collection (ATCC, Manassas, VA, USA) and cultured in Dulbecco's Modified Eagle's Medium (DMEM, ThermoFisher Scientific, Waltham, MA, USA) containing 10% fetal bovine serum (FBS, Biosera, Kansas City, MO, USA) and 1% penicillin/streptomycin (100 U/mL, ThermoFisher Scientific). H9C2 cells were treated with H_2_O_2_ (30 μM) for 2 h and subsequently cultured for 24 h. Primary cardiomyocytes were isolated from aged Rap1^+/+^ and Rap1^-/-^ mice (male, 12 months old) as described **26**. Briefly, mice were anesthetized and subsequently heparinized (0.5 mL heparin per mouse, 100 IU/mL, LEO Pharma, Denmark) to prevent coagulation of blood in the coronary arteries. Mouse hearts were collected quickly and immediately perfused with perfusion buffer, followed by perfusion with digestion buffer containing collagenase II (2.4 mg/mL, ThermoFisher Scientific). After digestion, the heart was minced and myocytes mechanically dispersed and filtered. The rod-shape primary cardiomyocytes were cultured and maintained on Matrigel-coated culture plates in Minimum Essential Medium Eagle (MEM, ThermoFisher Scientific) supplemented with 0.1% bovine serum albumin (BSA, Sigma-Aldrich), 1% penicillin/streptomycin and 2 mM Glutamine (Sigma-Aldrich). Subgroups of the primary cardiomyocytes were treated with or without PFTα (10 µM, 12 h) or WY14643 (selective PPARα agonist, 10 µM, 3 h, Cayman Chemical Company, Ann Arbor, MI, USA) and then subjected to the measurement of oxygen consumption rates (OCR). Another subgroup of the primary cardiomyocytes was treated with Nutlin 3a (10 µg/mL, a potent p53-MDM2 interaction inhibitor, Sigma-Aldrich) or DMSO as vehicle for 6 h, followed by RNA extraction and protein extraction for Q-PCR and Western blotting, respectively. All cells were cultured at 37 °C in a humidified atmosphere containing 5% CO_2_-95%O_2_.

### Seahorse analysis of oxygen consumption rate

Fatty acid oxidation in cardiomyocytes from aged Rap1^+/+^ and Rap1^-/-^ mice (male, 12 months old) was assessed as OCR in the presence of palmitate-BSA by using XF^e^24 respirometer platform (Seahorse Bioscience XF^e^24 Extracellular Flux Analyzer, Seahorse Bioscience, Billerica, MA, USA) as previously described 27. Briefly, isolated cardiomyocytes at a density of 15,000 cells per well were seeded onto Matrigel-coated Seahorse microplates with 500 µl XF Based Medium (Seahorse Bioscience) and incubated in 37 °C non-CO_2_ incubator for 1 h before loading into the XF^e^24 respirometer platform. The following compounds were injected sequentially into the culture plate wells: palmitate-BSA (0.5 mM palmitate-0.3% BSA), oligomycin (1 µM), carbonyl cyanide 4-(trifluoromethoxy) phenylhydrazone (FCCP, 1 µM) and rotenone/antimycin A (1 µM). Raw traces were normalized to basal respiration.

### DNA extraction

The genomic DNA of the heart from aged Rap1^+/+^ and Rap1^-/-^ mice (male, 12 months old) was extracted using the PureGenome^TM^ Tissue DNA extraction kit (Merck, St. Louis, MO, USA), according to the manufacturer's protocol. DNA concentration was quantified with the ultraviolet-visible spectrophotometer (NanoDrop 2000, ThermoFisher Scientific).

### Measurement of telomere length

Relative average telomere length was calculated by measuring the quantity of telomeric repeats to single-copy gene (T/S ratio) and determined by a real-time PCR approach 28. The sequence of the telomere-specific forward (tel1b), reverse (tel2b) primers and single-copy gene, acidic ribosomal phosphoprotein PO forward (36B4u) and reverse (36B4d) primers are listed in **Table 1**. The thermal profile to measure telomeres was 95 °C hot start for 10 min, followed by amplification rounds consisting of 30 cycles at 95 °C for 15 s and anneal-extension at 56 °C for 1 min. The 36B4 PCR was performed at 95 °C hot start for 10 min, followed by amplification rounds consisting 35 cycles at 95 °C for 15 s and anneal-extension at 60 °C for 1 min. The experiment was conducted using the StepOnePlus Real-Time PCR system (Applied Biosystems, Foster City, CA, USA).

### Quantitative Polymerase Chain Reaction

Total RNA was extracted from heart tissue of aged Rap1^+/+^ and Rap1^-/-^ mice and primary cardiomyocytes using RNAiso Plus (Takara, Shuzou, Japan) and complementary DNA was synthesized from equal amounts of RNA by reverse transcription using PrimeScript RT Reagent Kit (Takara) using oligodT primers, according to the manufacturer's instructions. Quantitative PCR was performed using SybrGreen Supermix (Takara) with specific primers (**Table 2**) in StepOnePlus Real-Time PCR system as previously described 29. The thermal profile was 95 °C for 15 s, followed by 40 cycles of denaturation at 95 °C for 3 s and anneal-extension at 60 °C for 31 s. β-actin was used as an internal control to normalize the mRNA level of target gene expression in all experiments.

### Nuclear extraction

The nuclear extraction of the heart tissue and cardiomyocytes was conducted using a commercially available nuclear extraction kit (ThermoFisher Scientific), according to the manufacturer's protocol. The protein concentration of the samples was determined with the Bicinchonic Acid assay (ThermoFisher Scientific).

### Western blotting

Heart and cell lysates were prepared using a lysis buffer (Cell Signaling Technology) supplemented with Protease Inhibitor Cocktail (Roche, Mannheim, Germany) and Phosphatase Inhibitor Cocktail (Roche). Equal denatured protein lysates from cells or heart tissues were separated by 8%-12.5% sodium dodecyl sulphate polyacrylamide gel electrophoresis and transferred onto polyvinylidene difluoride membranes, as previously described 30. The primary antibodies against p53 (1:1000), Acetyl-p53 (Lys379, 1:1000), γH2AX (1:1000), carnitine palmitoyltransferase 1α (CPT1α, 1:1000), Acetyl-CoA carboxylase (ACC, 1:1000), GAPDH (1:2000), Histone H3 (1:1000) were purchased from Cell Signaling Technology, as was horseradish peroxidase-conjugated anti-mouse (1:3000) or anti-rabbit (1:3000) secondary antibodies. The primary antibodies against long-chain specific acyl-CoA dehydrogenase (ACADL, 1:1000) and CD36 (1:2000) were purchased from Abcam (Cambridge, MA, USA). The primary antibodies against Rap1 (1:500), PPARα (1:1000), PGC1β (1:1000) and p21 (1:1000) were ordered from Santa Cruz Biotechnology and the anti-PGC1α antibody (1:1000) was ordered from Novus Biologicals (Littleton, CO, USA). The membrane was developed with Clarity ECL Western Blotting Detection Reagent (Bio-Rad) and subsequently placed on X-ray film for imaging. The optical densities of the immunoreactive bands were quantified using the ImageJ software.

### ATP assay

Whole heart tissue homogenates from aged Rap1^+/+^ and Rap1^-/-^ mice (male, 12 months old) were used to determine the levels of ATP using a commercial ELISA kit (ABclonal, Woburn, MA, USA), according to the manufacturer's instruction and normalized with protein concentration. The content of ATP is expressed as pmol/µg protein.

### Transmission electron microscopy for mitochondrial ultrastructure

Freshly harvested LV tissue from aged Rap1^+/+^ and Rap1^-/-^ mice (male, 12 months old) was processed for transmission electron microscopy. Briefly, blocks of LV myocardium (~1 mm^3^) were fixed with 2.5% glutaraldehyde in 0.1 M cacodylate buffer at 4 °C for 4 h and post-fixed with 1% osmium tetroxide at room temperature for 1 h, followed by dehydration, embedding in epoxy resin, and polymerization at 60 °C overnight, as previously described 31. Ultra-thin sections (100 nm) were stained with uranyl acetate and Reynold's lead citrate and photographed with a transmission electron microscope (CM100; Philips Electron Optics, Eindhoven, The Netherlands).

### Chromatin immunoprecipitation

Heart tissue from aged wildtype mice was minced and cross-linked by 1% formaldehyde for 20 min at room temperature, followed by addition of glycine (125 mM) to stop the cross-linking reaction. The tissue was lysed in a lysis buffer supplemented with Protease Inhibitor Cocktail (provided in the assay kit) and subsequently sonicated to generate DNA fragments. Total tissue lysates were then subjected to immunoprecipitation with protein A/G magnetic beads in the presence of primary p53 antibody (1:200) or mouse IgG control overnight at 4 °C. After washing by lysis buffer, the protein/DNA complex was eluted with elution buffer, followed by incubation at 62 °C for 2 h to reverse the cross-links of protein/DNA complex to free DNA. The DNA purification was performed by using commercial spin columns (Merck), according to the manufacturer's instruction. The DNA fragments were analysed by real time PCR using specific PPARα primers (**Table 3**).

### Measurement of plasma triglyceride

Plasma levels of triglycerides in aged Rap1^+/+^ and Rap1^-/-^ mice (male, 12 months old) were measured using commercial enzymatic kits (StanBio Laboratory, Boerne, TX, USA) as we described 32.

### Determination of protein expression in human heart tissue

Human myocardial specimens were collected from a subset of adult patients (n = 12) in Guangdong Provincial People's Hospital during the time of open heart surgery of mitral valve replacement, heart transplantation or donation after the approval by the ethic Review Board of Guangdong Provincial People's Hospital, and the obtaining of written informed consent from participants. The patients' cardiac specimens were collected and coded by two researchers (YH and JX) independently and were processed for protein assay by a third researcher (YC). The collected heart samples were immediately snap frozen in liquid nitrogen and stored at -80 °C for further protein determination (including Rap1, p53, PPARα, and other fatty acid metabolism-related enzymes) by Western blotting analysis. The patients were then divided into a young group ( < 40 years, n = 7) and a middle-age/aged group ( > 40 years, n = 5) according to age at the time of data analysis, and the levels of the respective protein expression in the middle-age/aged group were expressed as fold changes relative to the young group.

### Statistics

Results are presented as mean ± standard error of the mean (S.E.M.). Comparison between groups was carried out by two-tailed non-parametric Mann Whitney test, unpaired Student's t-test, or two-way ANOVA followed by Bonferroni test, where appropriate, using the GraphPad Prism 8.0 software (San Diego, CA, USA). Survival curve was developed using the Kaplan-Meier method with log-rank test. In all comparisons, a P value < 0.05 was accepted as statistical significance.

## Results

### Protein expression of Rap1 was reduced in the aged hearts of mouse and human and in H_2_O_2_-treated H9C2 cells

**Figure 1:**
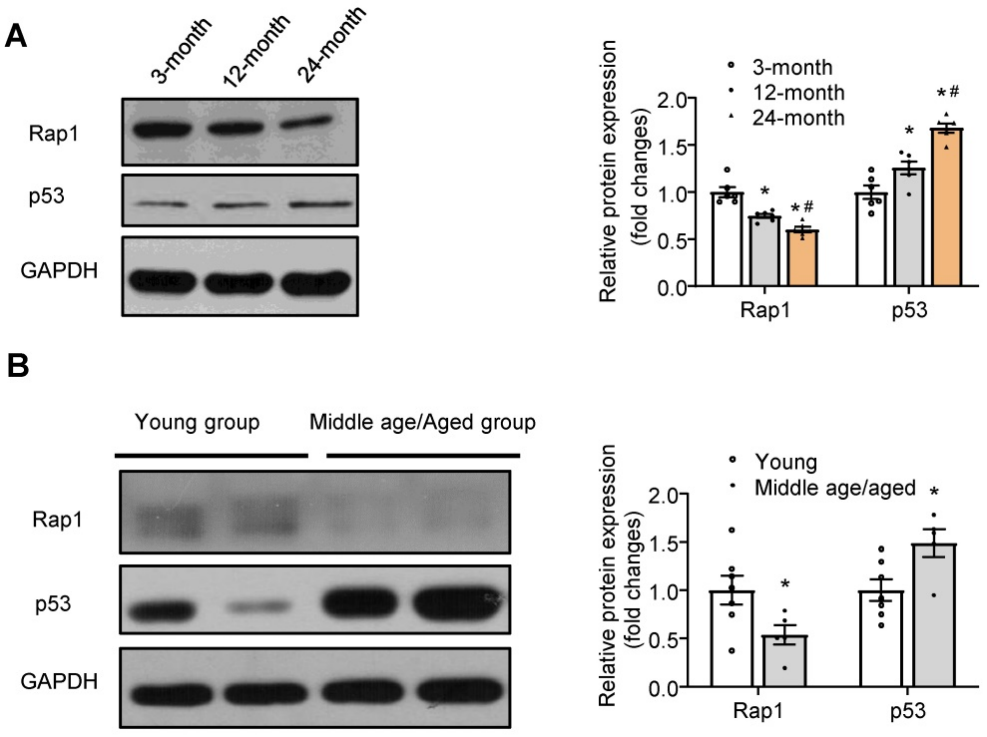
Protein expression of Rap1 was reduced with concomitantly enhanced p53 in the aged heart of mouse and human. (A) Representative Western blots of Rap1 and p53 expression in myocardium of mice at different ages (3-month, 12-month, 24-month). n = 6; (B) Representative Western blots of Rap1 and p53 in human myocardium with different age groups (young group, < 40 years, n = 7; middle age/aged group, > 40 years, n = 5). Protein presence of Rap1 and p53 was normalized to GAPDH. Data are shown as means ± S.E.M; ^*^P < 0.05 12-month or 24-month* vs*. 3-month; or middle age/aged group *vs*. young group; ^#^P < 0.05 24-month* vs*. 12-month.

To understand the relation of Rap1 with aging heart, we investigated its protein expression in mice of different ages. Rap1 was abundantly expressed in wildtype mouse hearts at 3 months of age, whereas it was reduced by 25% and 40% at 12 months and 24 months of age, respectively, indicating an age-dependent reduction of Rap1 protein in the heart during physiological cardiac aging (**Figure 1A**). Furthermore, an age-dependent increase in the protein expression of p53 (a cellular senescence marker) was observed in the myocardium of mouse at different ages, suggesting a co-relationship between Rap1 and p53 (**Figure 1A**). To evaluate the relation of Rap1 with aging human hearts, the protein presence of Rap1 and p53 were examined in the human myocardium from different age groups irrespective of the heart disease in these patients. Besides the significant difference in the age [young group (median age, 32) and middle age/aged groups (median age, 61)], there was no significant differences in body weight, blood pressure (systolic and diastolic) and LVEF between young and middle age/aged groups (**Table S1**). Consistently, significantly reduced Rap1 expression was also found in the aged human hearts, when compared with that in the hearts of young subjects (**Figure 1B**). In addition, the expression of p53 was significantly enhanced in aged human hearts (**Figure 1B**).

To further demonstrate the down-regulation of Rap1 with aging, we treated H9C2 cells with H_2_O_2_, a widely used stimulus for the induction of cell senescence through superoxide-induced DNA damage to mimic aging 33. Treatment of H9C2 cells with 30 μM of H_2_O_2_ for 2 h and subsequent culturing for 24 h successfully induced cells senescence, as evidenced by enhanced p53 expression (**Figure S1A**) without causing apparent cell injury (as evaluated by no significant change in the release of lactate dehydrogenase as compared to control) (**Figure S1B**). In parallel, a decrease in the protein expression of Rap1 was also evident (**Figure S1A**), indicative of co-occurrence of compromised Rap1 levels and aggravated cardiomyocyte senescence.

### Loss of Rap1 exacerbated cardiac hypertrophy, senescence and global cardiac performance

To understand the significance of reduced protein expression of Rap1 in the aging process, we investigated if the global constitutive Rap1^-/-^ mice would age faster than the wildtype counterparts. Rap1^-/-^ mice were born viable at the expected Mendelian ratios without apparent defects at 3 months old, suggesting an absence of embryonic lethality. However, Rap1^-/-^ mice started aging-associated phenotypes thereafter during their development as indicated by earlier hair graying starting at ~ 6 months of age, massive hair loss (**Figure S2A**) and reduced body weight (**Figure S2B**) at age 20 months when compared to age-matched wildtype mice. This is further supported by the novel object recognition tests, based on the tendency of rodents to preferentially explore a novel objective relative to a familiar object, in which aged Rap1^-/-^ mice exhibited a decline (51%) in novel object recognition at 24 h post sample stage relative to the wildtype mice (**Figure S2C**). In addition, the survival test on the male mice showed a progressive decline of lifespan in Rap1^-/-^ mice and median survival time in the Rap1^-/-^ mice (90.5 weeks) was significantly shorter as compared with wildtype counterparts (113.5 weeks) (**Figure S2D-E**).

**Figure 2:**
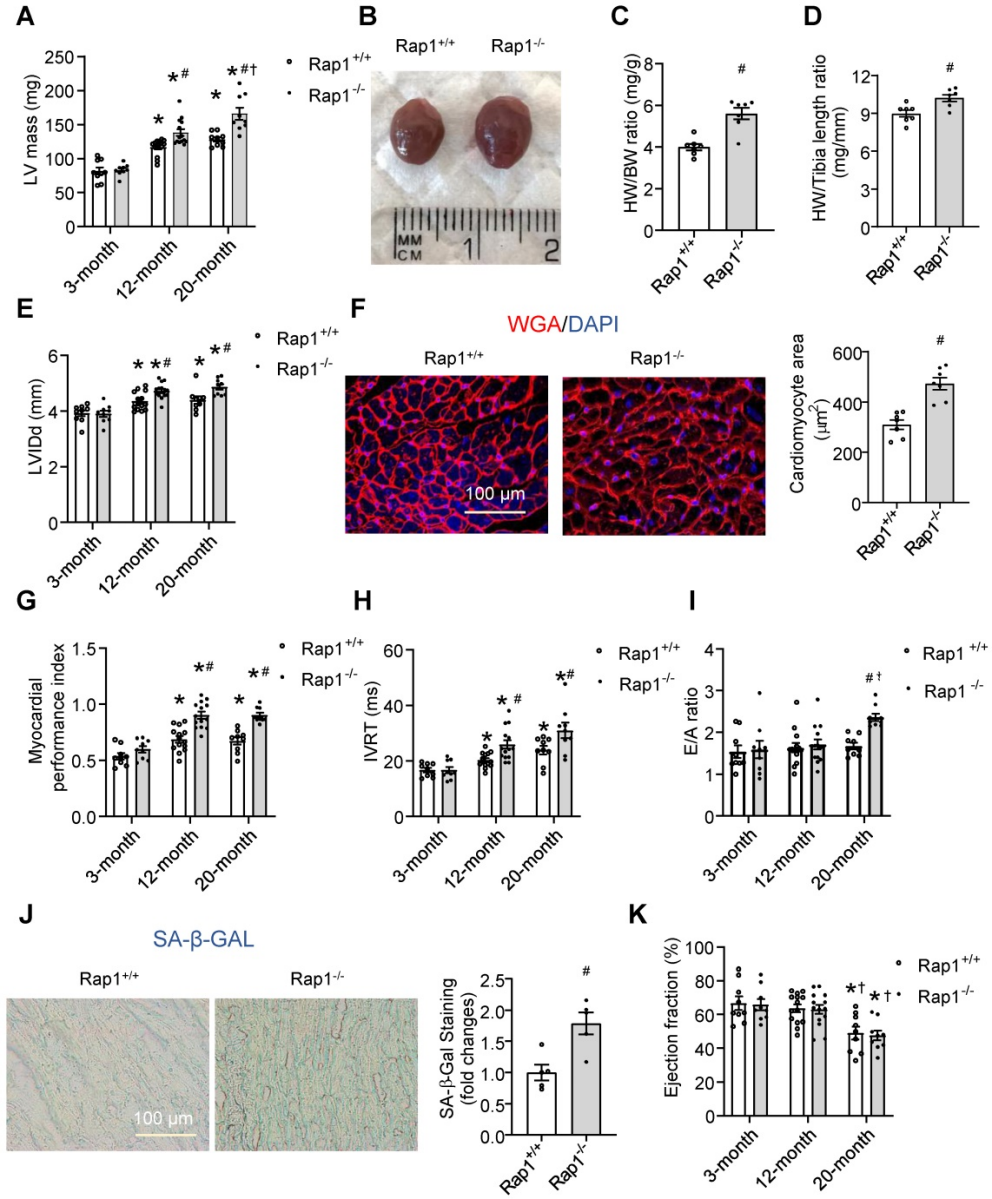
Loss of Rap1 exacerbated cardiac hypertrophy, senescence and global cardiac performance. Transthoracic echocardiography was performed to evaluate left ventricular mass (A) in Rap1^+/+^ and Rap1^-/-^ mice at different ages (3-month, n = 9; 12-month, n = 14; 20-month, n = 9). (B) Representative pictures from whole heart of Rap1^+/+^ and Rap1^-/-^ mice at 12-month. (C) Heart weight/body weight ratio and (D) heart weight/tibia length ratio of Rap1^+/+^ and Rap1^-/-^ mice at 12-month, n = 7; (E) Left ventricular end-diastolic diameter (LVIDd) in Rap1^+/+^ and Rap1^-/-^ mice at different ages (3-month, n = 9; 12-month, n = 14; 20-month, n = 9). (F) Representative pictures of wheat germ agglutinin staining and quantification of cardiomyocyte area (n = 7) in the heart of Rap1^+/+^ and Rap1^-/-^ mice at 20-month (n = 5). Myocardial performance index (MPI, G), isovolumic relaxation time (IVRT, H) and ratio of the early to late ventricular filling velocities (E/A ratio, I) in Rap1^+/+^ and Rap1^-/-^ mice at different ages (3-month, n = 9; 12-month, n = 14; 20-month, n = 9). (J) Representative pictures of SA-β-Gal-stained heart sections and quantification of SA-β-Gal-staining positive area in the heart of Rap1^+/+^ and Rap1^-/-^ mice at 20-month (n = 5). (K) Ejection fraction (EF) in Rap1^+/+^ and Rap1^-/-^ mice at different ages (3-month, n = 9; 12-month, n = 14; 20-month, n = 9). Magnification 400×: Scale: 100 μm. Data are shown as means ± S.E.M; ^*^P < 0.05 12-month or 20-month* vs*. 3-month; ^#^P < 0.05 Rap1^-/-^ *vs*. Rap1^+/+^;^ †^P < 0.05 20-month* vs*. 12-month.

To understand whether Rap1 deficiency would affect cardiac function, echocardiography follow-up was performed in Rap1^+/+^ and Rap1^-/-^ mice during their development from 3 months, 12 months to 20 months. Aged-dependent increases in the LV mass was observed in both types of the mice, of which the increase was more pronounced in the Rap1^-/-^ mice at 12 or 20 months of age relative to the age-matched wildtype mice (**Figure 2A**). Consistent with this, a marked increase in the heart size (**Figure 2B**), the heart weight/body weight ratio (**Figure 2C**) and the heart weight/tibia length ratio (**Figure 2D**) was observed in Rap1^-/-^ mice relative to wildtype counterparts at age of 12 months.

Similar to the alterations in LV mass, an age-dependent significant elevation in LVIDd was seen in both the wildtype and Rap1^-/-^ mice at the age of 12 months as compared to the respective values at 3 months. This elevation in LVIDd was maintained at significantly elevated levels at 20 months of age, and the elevations in LVIDd over time were more pronounced in Rap1^-/-^ mice than those in wildtype mice (**Figure 2E**). Meanwhile, the cross-sectional area of cardiomyocytes measured by WGA staining was significantly increased in the Rap1^-/-^ mouse heart at 20 months old relative to the age-matched wildtype counterparts (**Figure 2F**). Regarding cardiac function, we investigated MPI, IVRT and E/A ratio as factors of diastolic/systolic performance, SA-β-Gal activity as cardiac senescence and ejection fraction as an indicator of systolic function. The age-dependent increase in MPI was more pronounced in Rap^-/-^ mice than that in wildtype mice at 12 months of age, indicating reduced cardiac performance with Rap1 deficiency, and the level of MPI did not further increase at 20 months old (**Figure 2G**). Further, age-dependent increase in IVRT was significant in wildtype mice, which was further exacerbated in Rap1^-/-^ mice (**Figure 2H**). In contrast, the E/A ratio was unaffected by age throughout 12 months in either type of the mice, and continued to be preserved in Rap1^+/+^ mice at the age of 20 months. However, an increase in E/A ratio was evident in Rap1^-/-^ mice at age of 20 months relative to wildtype mice (**Figure 2I**). Meanwhile, the level of SA-β-Gal activity, an indicator of cardiac senescence, was significantly higher in aged Rap1^-/-^ mice hearts (20 months old) relative to that in the age-matched wildtype mice (**Figure 2J**), suggesting a worsening of cardiac senescence with Rap1 deficiency. However, the decrease in the EF% was not evident until 20 months of age in both types of the mice, in which a similar level of EF% was observed in both strains (**Figure 2K**), demonstrating an independent relation between systolic dysfunction and Rap1 deficiency. Thus, although lack of Rap1 did not significantly contribute to systolic dysfunction, an age-dependent reduction in the cardiac performance and increase in senescence was seen with Rap1 deficiency in mice.

### Loss of Rap1 shortened telomere and triggered DNA damage in the aging heart

**Figure 3:**
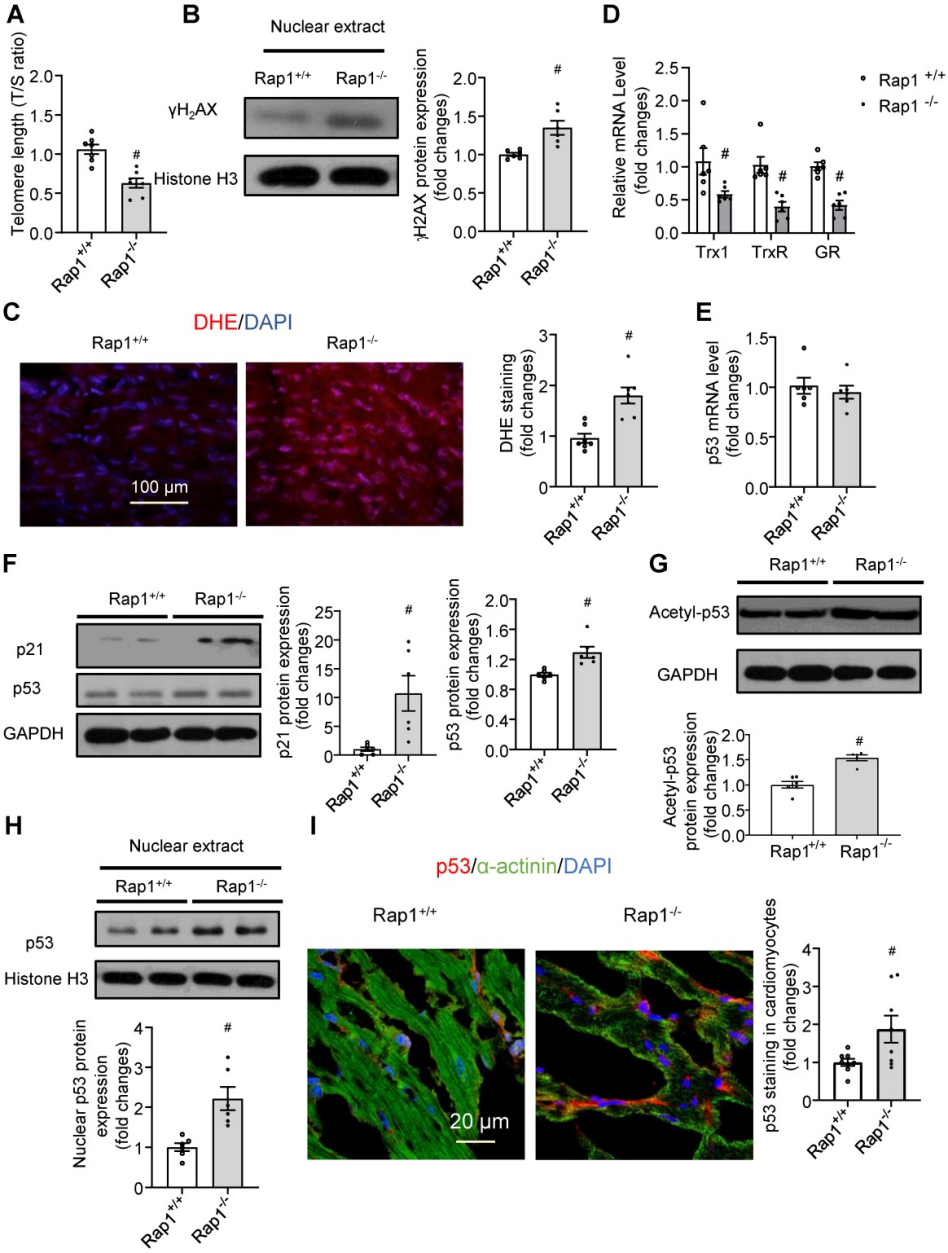
Loss of Rap1 led to shorter telomere and triggers DNA damage in the aging heart. In the heart of Rap1^+/+^ and Rap1^-/-^ mice at 12-month of age, (A) the ratio of telomeric repeats with single-copy gene (T/S ratio) n = 7; (B) Representative Western blots of nuclear γH2AX expression, n = 6; (C) Representative pictures of dihydroethidium (DHE) staining, n = 7; (D) mRNA expression of thioredoxin-1 (Trx1), thioredoxin reductase (TrxR) and glutathione reductase (GR), n = 6; (E) mRNA expression of p53, n = 6; Representative Western blots of p21, p53 (F), acetyl-p53 (G) and nuclear p53 (H) expression, n = 6. (I) Representative field of view and quantification of immunofluorescent staining of p53 (red), α-actinin (green) and nucleus (DAPI, blue) in adjacent heart sections. Magnification 630×, scale bars: 20 µm; n = 8. Protein presence of nuclear γH2AX and nuclear p53 was normalized to Histone H3. Protein presence of p21, p53 and acetyl-p53 was normalized to GAPDH. mRNA levels are expressed as fold changes against those mRNA expressions in the myocardium of Rap1^+/+^ mice. Data are shown as means ± S.E.M; ^#^P < 0.05 Rap1^-/-^ *vs*. Rap1^+/+^.

Short telomeres have been implicated in the pathobiology of different aging-related diseases, including cardiac aging 17,18. Given that Rap1 plays a crucial role in telomere length in the context of telomerase deficiency and that telomerase activity is reduced with aging 34, we performed quantitative polymerase chain reaction analysis to determine the telomere length in the non-proliferative heart tissues obtained from aged Rap1^+/+^ and Rap1^-/-^ mice at 12 months of age (mimics the status of defective telomerase relative to mice at 2-3 months of age). The T/S ratio was significantly reduced in the hearts from aged Rap1^-/-^ mice (**Figure 3A**), indicating that loss of Rap1 resulted in shorter telomere lengths in the heart.

Telomere shortening and uncapped telomeres activates DNA damage response, leading to the rapid phosphorylation of histone H2AX at serine 139 (γH2AX), which is recognized as a marker of DNA double strand breaks 35. Upon DNA damage, activation of p53 and its canonical target p21 leads to cell cycle arrest and cell senescence, eventually resulting in tissue failure and age-dependent pathologies 36,37. We thus further addressed whether the shorter telomere length in the hearts of aged Rap1^-/-^ mice is associated with severe DNA damage, ROS generation and DNA damage response. At the middle-aged time point (12 months old), deletion of Rap1 in mice significantly increased the cardiac nuclear levels of γH2AX (**Figure 3B**). Furthermore, aged Rap1^-/-^ mice exhibited greater ROS generation (**Figure 3C**) and reduced cardiac mRNA expression of anti-oxidant thiol-redox enzymes, including thioredoxin-1, thioredoxin reductase and glutathione reductase, as compared to the wildtype counterparts (**Figure 3D**). Although p53 mRNA levels were not altered (**Figure 3E**), significantly increased p21 and p53 protein expression was confirmed by immunoblot in the hearts of aged Rap1^-/-^ mice relative to age-matched wildtype counterparts (**Figure 3F**). Furthermore, acetylation of p53 (Lys-379, indicator for nuclear translocation) and nuclear p53 expression in the heart were also significantly enhanced in aged Rap1^-/-^ mice when compared to wildtype mice (**Figure 3G-H**). To validate that p53 localized predominately within cardiomyocytes, double immunofluorescence staining [p53 (red), α-actinin (cardiomyocyte specific marker, green)] was performed. The merged images showed that p53 co-localized majorly in cardiomyocytes within and outside of the nuclear in both strains. Additionally, the p53 expression within cardiomyocytes was significantly higher in the heart sections of aged Rap1^-/-^ mouse when compared with wildtype counterparts (**Figure 3I**). The collective phenotypic profile observed is consistent with expectations of severe cardiac senescence and aging in Rap1^-/-^ mice.

### Loss of Rap1 reduced fatty acid metabolism with aging *via* PPARα

**Figure 4:**
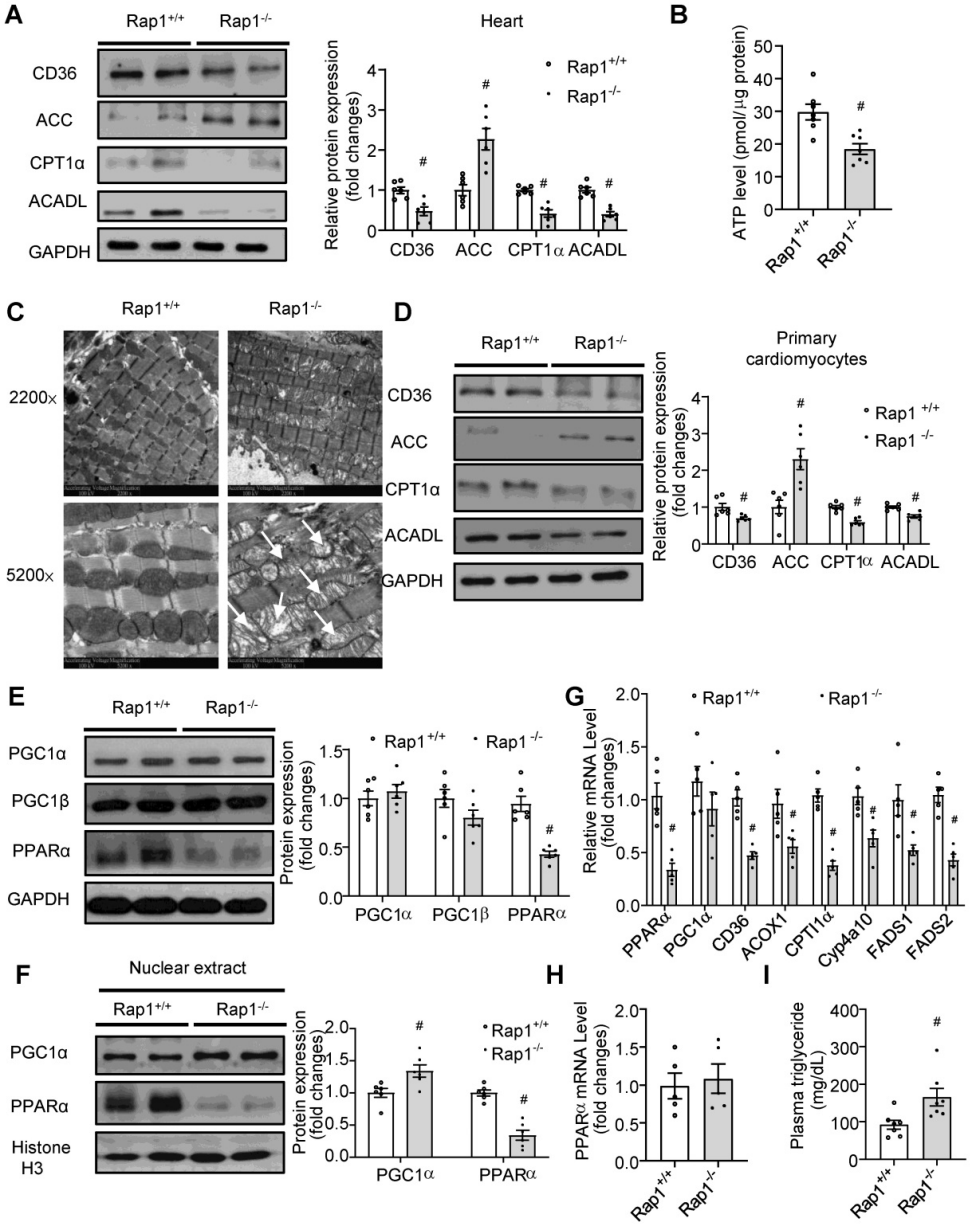
Loss of Rap1 reduced fatty acid metabolism with aging *via* PPARα. (A) Representative Western blots of CD36, ACC, CPT1α and ACADL expression (n = 6) and (B) ATP production (n = 7) in the heart of Rap1^+/+^ and Rap1^-/-^ mice at 12-month of age; (C) Representative electron micrographs of mitochondria in the hearts of Rap1^+/+^ and Rap1^-/-^ mice at 12-month of age with low (2200×) and high (5200×) magnifications. White arrow indicates more cristae fragmentation and vacuolization in Rap1^-/-^ mice. (D) Representative Western blots of CD36, ACC, CPT1α and ACADL expression (n = 6) in the primary cardiomyocytes from the hearts of Rap1^+/+^ and Rap1^-/-^ mice at 12-month of age. Representative Western blots of PGC1α, PGC1β and PPARα expression (E) and nuclear PGC1α and PPARα expression (F) in the hearts of Rap1^+/+^ and Rap1^-/-^ mice at 12-month of age, n = 6. Protein presence of CD36, ACC, CPT1α, ACADL, PGC1α, PGC1β and PPARα was normalized to GAPDH. Protein presence of nuclear PGC1α and PPARα was normalized to Histone H3. (G) mRNA expression of PPARα, PGC1α, CD36, ACOX1, CPT1α, Cyp4a10, FADS1 and FADS2 in the hearts of Rap1^+/+^ and Rap1^-/-^ mice at 12-month of age, n = 5. (H) mRNA expression of PPARα in the hearts of Rap1^+/+^ and Rap1^-/-^ mice at 3-month of age, n = 5. mRNA levels are expressed as fold changes against those mRNA expressions in the myocardium of Rap1^+/+^ mice. (I) Plasma triglyceride level in the Rap1^+/+^ and Rap1^-/-^ mice at 12-month of age, n = 7. Data are shown as means ± S.E.M; ^#^P < 0.05 Rap1^-/-^ *vs*. Rap1^+/+^.

With aging, the heart exhibits impaired metabolic flexibility, including reduced fatty acid metabolism and ATP generation 4,5. However, whether Rap1 has a role in this reduction remains unclear. Thus, we first examined the expression of key enzymes involved in fatty acid metabolism and the electron transport chain (ETC). The expression of CD36 (a sarcolemmal fatty acid transporter), CPT1α (involved in mitochondrial fatty acid uptake) and ACADL (an enzyme of fatty acid oxidation) were decreased while ACC (which catalyzes the production of malonyl-CoA, which prevents the fatty acids to enter mitochondria) increased in the Rap1^-/-^ mouse hearts (**Figure 4A**), resulting in reduced levels of ATP (**Figure 4B**) as compared to age-matched wildtype at 12 months of age. In contrast, the expression of mitochondrial complex I-V was unaffected by the lack of Rap1 (**Figure S3**). We then checked if Rap1 could affect mitochondrial structure. Indeed, an accumulation of enlarged, pleomorphic mitochondria containing cristae fragmentation, vacuolization and disrupted external membranes was seen in the aged Rap1^-/-^ mouse hearts at 12 months of age when compared to age-matched wildtype counterparts (**Figure 4C**). These results indicate a specific role of Rap1 on mitochondrial fatty acid metabolism rather than mitochondrial ETC. This is further supported by the enhanced protein expression of ACC and decreased protein expression of CD36, CPT1 α and ACADL in primary cardiomyocytes isolated from aged Rap1^+/+^ and Rap1^-/-^ mice at 12 months of age (**Figure 4D**).

As PPARα and PGC1α are the important transcriptional factors mediating fatty acid metabolism, we wanted to understand whether PPARα/PGC1α could have an association with Rap1. Strikingly, the total expression of PPARα, rather than PGC1α and PGC1β, was reduced in the aged Rap1^-/-^ mice (**Figure 4E**). More importantly, markedly reduced levels of nuclear PPARα, in contrast to a significantly increased level of nuclear PGC1α, was observed in the aged Rap1^-/-^ mice hearts relative to the age-matched wildtype counterparts (**Figure 4F**). Thus, PPARα, rather than PGC1α, may be associated with Rap1 in contributing to the impaired fatty acid metabolism in the aged Rap1^-/-^ mice hearts. Indeed, not only PPARα but also its downstream transcriptional targets, including CD36, ACOX1, CPT1α, Cyp4a10, FADS1 and FADS2 **39,** were reduced at the mRNA level in the aged Rap1^-/-^ mice hearts (**Figure 4G**). Of note, the mRNA level of myocardial PPARα was unaltered between the young Rap1^+/+^ and Rap1^-/-^ mice (3 months old) (**Figure 4H**), which strongly supported an age-dependent positive relation between Rap1 and PPARα. Furthermore, plasma triglyceride level was increased in the aged Rap1^-/-^ mice (**Figure 4I**), corroborating that loss of Rap1 may suppress PPARα and downstream enzymes to impair fatty acid metabolism.

### p53 mediated the effect of Rap1 loss on fatty acid metabolism and cardiac aging

**Figure 5:**
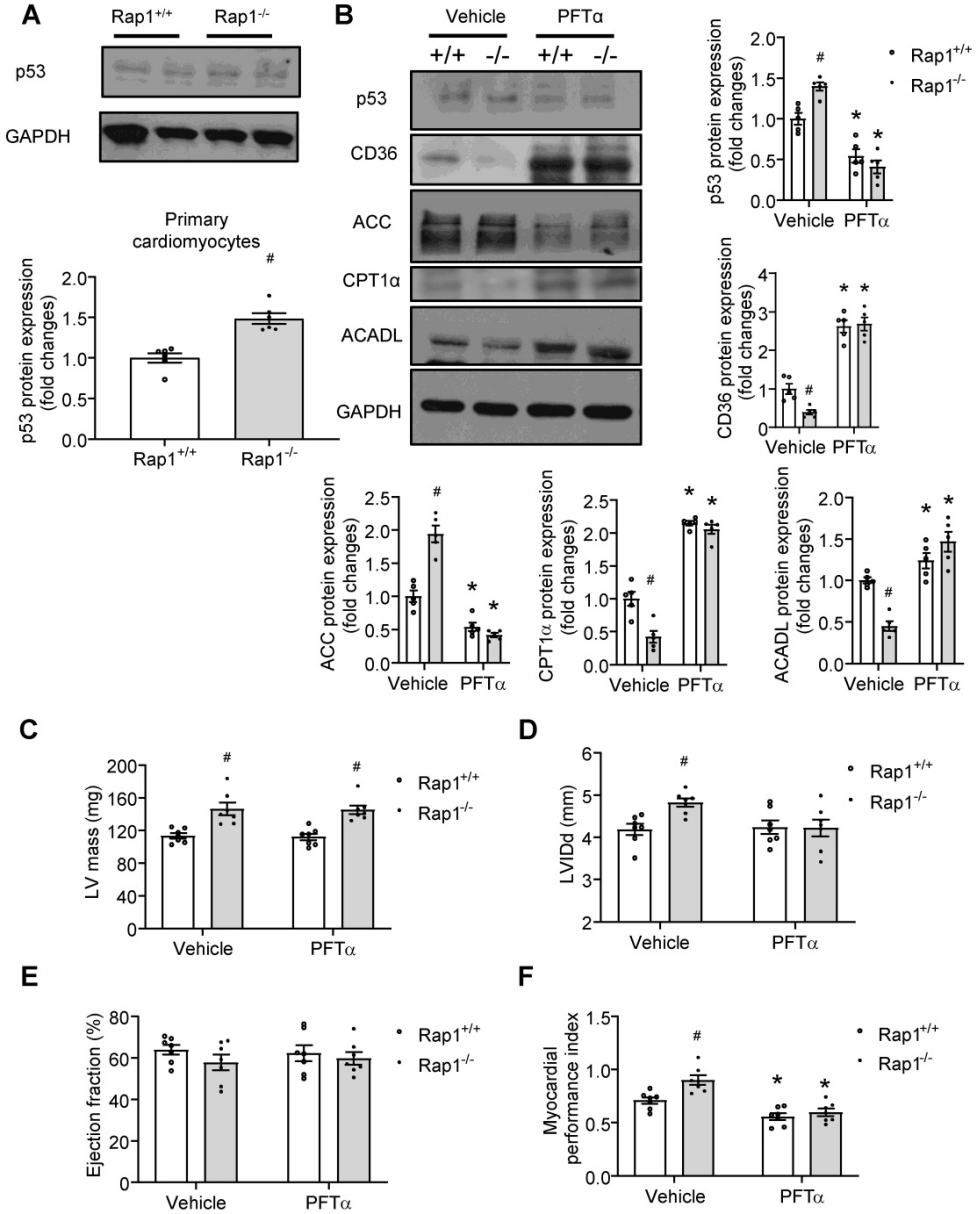
p53 mediates the effect of Rap1 loss on fatty acid metabolism and cardiac aging. (A) Representative Western blots of p53 expression in primary cardiomyocytes from the hearts of Rap1^+/+^ and Rap1^-/-^ mice at 12-month of age, n = 6. (B) Representative Western blots of p53, CD36, ACC, CPT1α and ACADL expression in the hearts of Rap1^+/+^ and Rap1^-/-^ mice at 12-month of age, with or without treatment of PFTα (1.1 mg/kg/day, 6 weeks), n = 5. Protein presence of p53, CD36, ACC, CPT1α and ACADL was normalized to GAPDH. Transthoracic echocardiography was performed to evaluate left ventricular mass (C), left ventricular end-diastolic diameter (LVIDd, D), ejection fraction (EF, E) and myocardial performance index (MPI, F) in Rap1^+/+^ and Rap1^-/-^ mice at 12-month of age, with or without treatment of PFTα, n = 7. Data are shown as means ± S.E.M; ^*^P < 0.05 PFTα* vs*. Vehicle; ^#^P < 0.05 Rap1^-/-^ *vs*. Rap1^+/+^.

Our data showed that p53 was activated in aged Rap1^-/-^ mice hearts (**Figure 3F-H**). Given that cardiac-specific ablation of p53 significantly up-regulated the genes essential for fatty acid oxidation, transportation, and synthesis in the heart 19, we proposed that a relationship exists between p53-Rap1-PPARα that may present in aged mice hearts that impact on the regulation of fatty acid metabolism. To determine this, we first checked the status of p53 protein and confirmed that the expression of p53 was increased in the primary cardiomyocytes of aged Rap1^-/-^ mice relative to the age-matched wildtype counterparts (**Figure 5A**). Then we investigated the effect of inhibiting p53 on basal metabolism in primary cardiomyocytes from aged wildtype mice (12 months old) by measuring OCR with the XF^e^24 extracellular flux analysis. A selective p53 inhibitor, PFTα (10 µM, 12 h) significantly enhanced the mitochondrial basal respiration by almost 3-fold (**Figure S4**) in the isolated cardiomyocytes. We then tested whether pharmacological inhibition of p53 could alleviate the impaired fatty acid metabolism, thereby attenuating cardiac aging *in vivo.* To do this we treated the aged Rap1^+/+^ and Rap1^-/-^ mice (12 months old) with PFTα (1.1 mg/kg/day, i.p.) for 6 weeks. Treatment with PFTα significantly reduced p53 expression in both Rap1^+/+^ and Rap1^-/-^ mice hearts (**Figure 5B**). Moreover, treatment with PFTα in aged Rap1^-/-^ mice significantly attenuated the protein expression of cardiac ACC and enhanced the cardiac presence of CD36, CPT1α and ACADL to levels similar to what is seen in the Rap1^+/+^ cardiac lysates (**Figure 5B**). This suggests that inhibition of p53 in aged Rap1^-/-^ mice can improve cardiac fatty acid metabolism *via* altering the expression of the related metabolic enzymes.

We also wanted to check if p53-mediated alterations in fatty acid metabolism could have beneficial effects on LV mass, LVIDd, EF% and cardiac performance. However, inhibition of p53 induced neither alterations on LV mass (**Figure 5C**), LVIDd (**Figure 5D**) nor on EF% (**Figure 5E**) in the aged Rap1^-/-^ mice hearts. In contrast, impaired cardiac performance, as indicated by the higher MPI in the aged Rap1^-/-^ mice hearts, was restored to a similar level in Rap1^+/+^ mice hearts (**Figure 5F**), indicating that inhibition of p53 improves cardiac performance independently without affecting LV mass.

### p53 directly suppressed the transcription of PPARα

**Figure 6:**
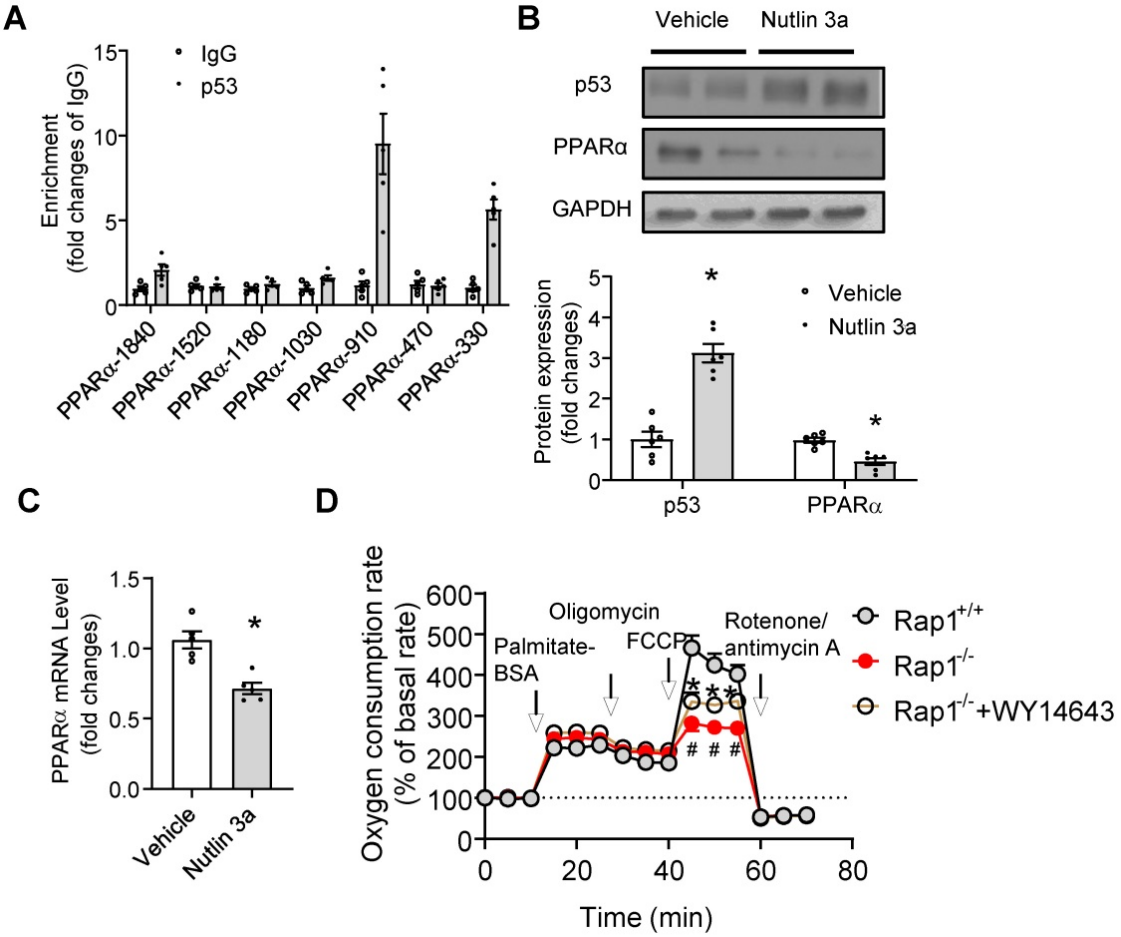
p53 directly suppresses transcription of PPARα. (A) Chromatin immunoprecipitation (ChIP) showing p53 binding on the promoters of PPARα at indicated sites, n = 5; (B) Representative Western blots of p53 and PPARα in primary cardiomyocytes from the heart of Rap1^+/+^ mice at 12-month of age, with or without treatment of Nutlin 3a (10 µg/mL, 6 h). Protein presence of p53 and PPARα was normalized to GAPDH. n = 6; (C) mRNA expression of PPARα in primary cardiomyocytes from the heart of Rap1^+/+^ mice at 12-month of age, with or without treatment of Nutlin 3a. mRNA levels were expressed as fold changes against that mRNA expression in the primary cardiomyocytes without treatment of Nutlin 3a, n = 5; (D) Oxygen consumption rates (OCR) was measured under basal, palmitate-BSA (0.5 mM), oligomycin (1 µM), FCCP (1 µM) and rotenone/antimycin A (1 µM)-stimulated conditions at indicated time points in the primary cardiomyocytes from the hearts of Rap1^+/+^ and Rap1^-/-^ mice at 12-month of age, with or without treatment of WY14643 (10 µM, 3 h). Data are shown as means ± S.E.M; ^#^P < 0.05 Rap1^-/-^ *vs*. Rap1^+/+^; ^*^P < 0.05 Nutlin 3a* vs*. Vehicle or Rap1^-/-^+WY14643 *vs*. Rap1^-/-^.

The fact that enhanced p53 expression and reduced cardiac mRNA and protein expression of PPARα was independent of PGC1α/β in aged Rap1^-/-^ mice prompted us to explore the possible direct links between p53 activation and PPARα regulation on multiple levels. Firstly, at the level of gene promoter, *in silico* promoter analysis was employed to explore the possibility of direct binding between p53 and the PPARα promoter, since several putative p53 response elements in the promoter region of the PPARα gene have been identified. Chromatin immunoprecipitation analysis demonstrated that there was significant enrichment of p53 to the PPARα fragments containing p53 binding elements at the location of -910 (~ 8 fold) and -330 (~ 6 fold), but not at -1840, -1520, -1180, -1030, -470 (**Figure 6A**). We then tested whether pharmacological activation of p53 can suppress PPARα. We treated the wildtype primary cardiomyocytes with Nutlin 3a (10 µg/mL, 6 h), a small molecule MDM2 inhibitor that blocks the MDM2-p53 interaction and activates p53 40. Treatment with Nutlin 3a resulted in significant induction of p53 expression in cardiomyocytes (**Figure 6B**), accompanied by reduction of mRNA and protein expression of PPARα (**Figure 6B and C**), indicating that activated p53 suppressed the expression of PPARα. In addition, we examined whether pharmacological activation of PPARα could restore the impaired fatty acid metabolism seen in the aged Rap1^-/-^ mice hearts. Fatty acid-mediated mitochondrial respiration was measured in primary cardiomyocytes from hearts of aged Rap1^+/+^ and Rap1^-/-^ mice, with or without the presence of WY14643 (selective PPARα agonist, 10 µM, 3 h), by using XF^e^24 extracellular flux analyser. The results showed that there was no change in the palmitate-BSA induced OCR among groups, but FCCP-induced OCR (which indicates the maximal respiratory capacity) was significantly attenuated in the aged Rap1^-/-^ cardiomyocytes when compared with the wildtype counterparts, while WY14643 pre-treatment partially restores FCCP-induced OCR in Rap1^-/-^ cardiomyocytes (**Figure 6D**). Taken together, these results demonstrated that activated p53 suppressed the expression of PPARα and led to impaired mitochondrial fatty acid metabolism, which was associated with a speeding up of the cardiac aging in Rap1^-/-^ mice.

### Impaired PPARα signaling in human myocardium with aging

**Figure 7:**
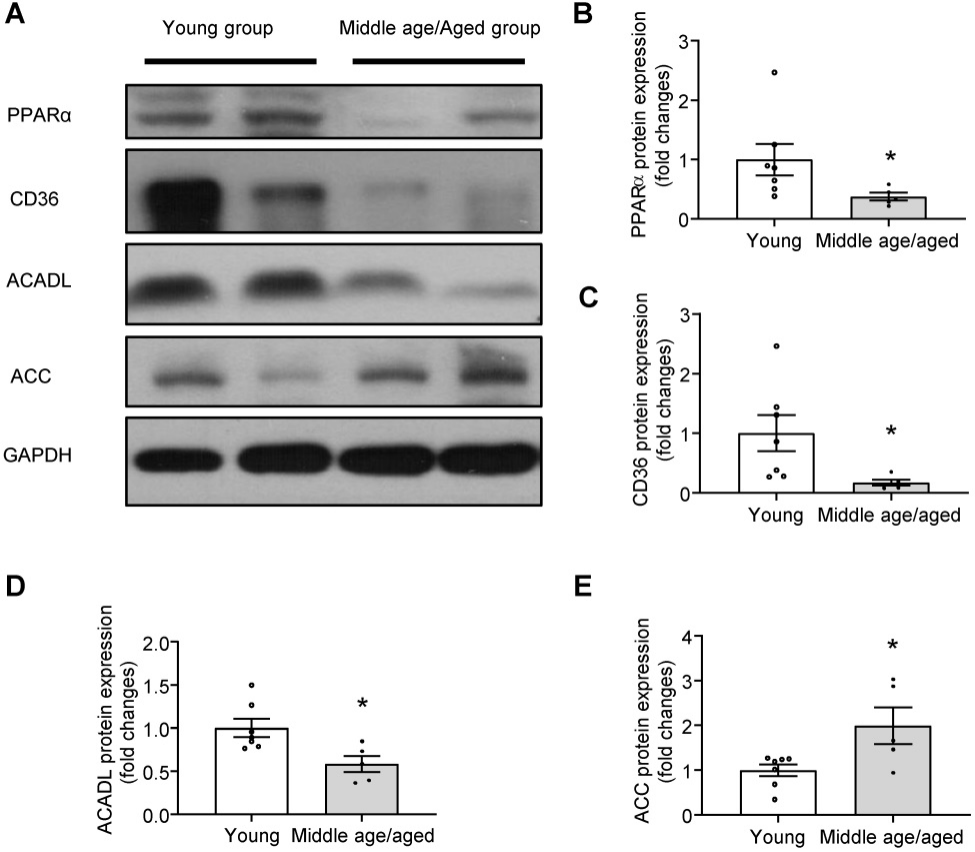
Impaired PPARα signaling in human myocardium with aging. (A) Representative Western blots and quantification of PPARα (B), CD36 (C), ACADL (D) and ACC (E) in human myocardium with different age groups (young group, n = 7; middle age/aged group, n = 5). Protein presence of PPARα, CD36, ACADL and ACC was normalized to GAPDH. Data are shown as means ± S.E.M; ^*^P < 0.05 middle age/aged group *vs*. young group.

To evaluate the relevance of the hypothesis tested in a murine model to that of human hearts, the protein presence PPARα and fatty acid metabolism-related enzymes were examined in the human myocardium from different age groups. Consistently, the expression of PPARα was markedly reduced in aged human hearts (**Figure 7A-B**). Furthermore, key enzymes in fatty acid metabolism, such as the expression of CD36, ACADL were significantly decreased (**Figure 7A, C-D**) and the expression of ACC was significantly increased (**Figure 7E**) in aged human hearts when compared with young groups. Given the reduced Rap1 and increased p53 expression in aged human myocardium, these observations suggested that the telomere associated Rap1 may be a crucial player in human cardiac aging *via* regulating p53/PPARα-dependent fatty acid metabolism.

## Discussion

We have several novel findings in the present study. First, Rap1^-/-^ mice showed progressive decreases in both median survival and mean lifespan when compared to wildtype mice. Second, aged Rap1^-/-^ mice displayed more pronounced aging-associated phenotypes. This included aggravated aging-related cardiac structural changes (increased LV wall thickness, LV mass) and altered cardiac performance (increased MPI) as well as greater cardiac senescence. Also, aged Rap1^-/-^ mice displayed shorter telomere length, greater DNA damage, abnormalities in the mitochondrial ultrastructure and reduced fatty acid metabolism. Importantly, we found that increased levels of p53 in aged mice with Rap1 deficiency was responsible for the reduced functional expression of PPARα *via* binding to the promoter region of the PPARα gene. Furthermore, in the aged human hearts, the expression of Rap1 was reduced concomitant with enhanced p53, reduced PPARα and altered fatty acid metabolic enzymes, including reduced CD36, ACADL and increased ACC, when compared to young groups. These findings provide direct evidence that telomere associated Rap1 plays an important role in the pathology of cardiac aging.

Telomere shortening and dysfunction has been involved in aging-related pathologies 17,18. However, controversy exists regarding the role of Rap1 in telomere length control 16,41,42. In human fibrosarcoma cells HTC75, Rap1 knockdown exhibited telomere elongation phenotype, indicating that mammalian Rap1 may negatively control telomere length 41. In other human cell lines, including Hela, HT1080, HTC116, ARPE-19, Rap1 deficiency did not alter the telomere length dynamic 42. In a recent study, Martinez *et al.* reported that Rap1 deficiency alone does not impact telomere length, but its deficiency leads to shorter telomere in intestine and blood cells in the context of telomerase deficiency 16. Given that telomerase activity reduces during aging 34, we speculated that with aging, deletion of mammalian Rap1 in the presence of aging associated reduction of telomerase activity may lead to a shorter telomere in the heart. Indeed, in the current study, we found that deficiency of Rap1 reduced telomere length with aging and decreased both median survival and mean lifespan in mice. It has been shown that cardiac aging and the associated cardiac functional decline in the mouse closely recapitulates human aging and function as significant predictors of mortality in mice 43. Indeed, in our study, Rap1^-/-^ mice displayed cardiac senescence and age-dependent alterations in LV structure, and a decline in cardiac performance which can give rise to their reduced lifespan. In particular, our evidence of a shorter telomere length and increased γH2AX in aged Rap1^-/-^ mice hearts provides a strong indication that Rap1 deletion accelerates telomere attrition, thereby resulting in DNA damage. Also, aging related DNA damage can explain the abnormalities in the mitochondrial ultrastructure seen in the Rap1^-/-^ mouse hearts. In line with this, others have shown that although cardiomyocytes are not subjected to repeated end-replication problem-associated telomere shortening, human and murine cardiomyocytes acquire a senescent-like phenotype characterised by persistent DNA damage at telomere regions 44.

Fatty acid metabolism plays an important role in maintaining cardiac performance and function. Taking into consideration that functional expression of p53 was increased in parallel with a reduced cardiac fatty acid metabolism, specifically reduced functional expression of PPARα in the aged Rap1^-/-^ mouse heart, we wanted to understand whether an interplay between Rap1, p53 and PPARα existed by investigating if increased p53 is related to reduced cardiac fatty acid metabolism through direct modulating functional PPARα. It has been suggested that p53, a molecule that modulates cellular senescence at different levels, could be an effector of telomere dysfunction *via* DNA damage response 38. Increased functional expression of p53 (total, nuclear p53 and acetyl-p53) without a change in p53 mRNA in the aged Rap1^-/-^ mice hearts is indicative of post-translational regulation of p53, which is mainly associated with increased acetyl-p53, thereby enhancing the stability of p53 to preserve nuclear and total level of p53. This notion is further supported by other studies that p53 acetylation promotes its stability by excluding ubiquitination-mediated protein degradation 45, 46. The enhanced p53 signaling serves as molecular underpinnings for the accelerated aging-related cardiomyopathy in aged Rap1^-/-^ mice. In line with this, in the telomerase reverse transcriptase knockout mice, that exhibit telomere shortening and a lack of telomerase activity, p53 deficiency results in a 30% improvement of fractional shortening in low-dosage doxorubicin-induced cardiomyopathy when compared with controls 38. This suggests that telomere dysfunction aggravates cardiac dysfunction through activation of p53 signaling. The prominent role of p53 in driving cardiac failure/degeneration is further supported by the findings that the increase in p53 in the heart was coincident with aging and heart failure 47,48. Cardiac-specific deletion of Mdm4, a negative regulator of p53, resulted in the development of dilated cardiomyopathy in mice through increased apoptosis and senescence, which provided further support that enhanced p53 signaling accelerated cardiac dysfunction 49.

To address whether p53 is associated with cardiac fatty acid metabolism, the aged Rap1^-/-^ mice were treated with an inhibitor of p53. Of interest, inhibiting p53 in the aged Rap1^-/-^ mice significantly improved the protein expression of key enzymes in cardiac fatty acid metabolism. The same also holds true in primary cardiomyocytes isolated from the aged wildtype mice, in which, inhibiting p53 significantly enhanced the mitochondrial basal respiration, as indicated by the elevated oxygen consumption rate. This is in consistent with additional roles of p53 as demonstrated by others, including the classical p53-mediated apoptosis pathway 50, the p53-dependent mitochondrial dysfunction pathway in the aging heart 38. Although LV mass and LVIDd were unaltered, inhibiting p53 reduced the MPI in the aged Rap1^-/-^ mice hearts, suggesting that p53 has a cardiac role at the level of cardiac fatty acid metabolism and cardiac performance, rather than the level of cardiac structure. Given the fact that cardiac-specific deficiency of p53 in mice led to upregulation of PPARα and its co-activator PGC1α 19 and thePPARα/PGC1α complex controls the expression of genes encoding enzymes involved in cardiac fatty acid uptake and oxidation 20,51, it is possible that p53 modulates cardiac fatty acid oxidation in Rap1^-/-^ mouse heart through a mechanism related to PPARα or PGC1α?

Emerging evidence demonstrates that p53 can directly bind to the promoter of PGC1α and repress its expression at a transcriptional level 38. However, we found no change in mRNA and protein expression of PGC1α between aged Rap1^+/+^ and Rap1^-/-^ mice, while the expression of PPARα and its targeted fatty acid metabolism related genes were significantly reduced in the aged Rap1^-/-^ mice hearts. These results implicate that, although the involvement of PGC1α can be rule out, PPARα may be a major downstream effector of p53 in the heart. Indeed, our study is the first to demonstrate that p53 can directly bind to the promoter of PPARα and repress its expression at a transcriptional level. This notion is further supported by other studies, in which aging-related cardiomyopathy is associated with reduced cardiac levels of PPARα 52. The reduction of PPARα activiation occurred in 20 months old rats, acompanied with accelerated cardiac aging, while treatment with atorvastatin increased PPARα expression and slowed down cardiac aging (including reduced cardiac hypertrophy, collagen deposition, oxidative stress and β-galactosidase activity 53) suggesting the importance of PPARα inhibition in cardiac aging. Also, PPARα deficiency in mice resulted in cardiac dysfunction that was accompanied with structural abnormalities in the mitochondria, increased oxidative stress, myocardial damage and fibrosis in the heart 54, 55. It is worthy of note that Rap1 has been suggested to work as a transcriptional factor and directly bind to the PPARα loci in the liver, and regulate the transcription of PPARα 11. However, the mRNA level of PPARα in the heart was unaltered between the young Rap1^+/+^ and Rap1^-/-^ mice, indicating that Rap1 deficiency-mediated reduction of PPARα expression in the aged mouse heart is p53-dependent, given the enhanced p53 signaling observed in aged Rap1^-/-^ mice hearts. To our knowledge, we are the first to demonstrate that accelerated cardiac aging due to the deletion of Rap1 is associated with an enhanced p53 signaling to suppress gene expression of PPARα. The clinical relevance of this mechanism is that the reduced expression of Rap1 and PPARα with an enhanced p53 was also observed in aged human hearts.

One of the limitations to note is that, Rap1 knockout mice are a global knockout and not a cardiac-specific knockout. This, therefore, cannot exclude the interfering effect of Rap1 knockout on non-cardiomyocytes. However, our *in vivo* findings were buttressed by *in vitro* studies with primary cardiomyocytes and double immunostaining experiments in heart sections. Furthermore, to confirm whether or not restoration of cardiac Rap1 levels may rejuvenate the cardiac function of aged hearts, the recombinant adeno-associated viruses (serotype 9) containing Rap1 and cardiac troponin T promoter can be used to treat the mice in future studies. In summary, the present study indicates that critical features of the cardiac aging and senescent phenotype are recaptitulated in the aged Rap1^-/-^ mice. With aging, Rap1 deficiency may exacerbate telomere attrition, which actives p53 and thereby suppresses PPARα expression. Reduced PPARα expression leads to impaired mitochondrial fatty acid metabolism (uptake and oxidation), reduced ATP generation and eventually results in compromised cardiac functional and structural changes. The clinical relevance of these findings was supported by the demonstration of reduced Rap1 expression, enhanced p53 presence and suppressed PPARα level in the aged human myocardium when compared with a young group. The present study forges a link to connect the most conserved telomere-associated protein Rap1 to cardiac aging and metabolic changes through the p53/PPARα signaling circuit. From a clinical translation point of view, the study lends credence to the possiblity of using Rap1/p53/PPARα signaling as therapeutic targets for treatment and intervention in the pathogenesis of cardiac aging.

## Supplementary Material

Supplementary figures and table.

## Figures and Tables

**Table 1:** Primers used in the measurement of telomere length in myocardium of mice

Gene name	Gene sequence 5'→3'
tel1b	CGGTTTGTTTGGGTTTGGGTTTGG GTTTGGGTTTGGGTT
tel2b	GGCTTGCCTTACCCTTACCCT TACCCTTACCCTTACCCT
36B4u	ACTGGTCTAGGACCCGAGAAG
36B4d	TCAATGGTGCCTCTGGAGATT

Abbreviations: 36B4u: acidic ribosomal phosphoprotein PO forward; 36B4d: acidic ribosomal phosphoprotein PO reverse; tel1b: telomere-specific forward; tel2b telomere-specific reverse.

**Table 2:** Primers used in quantitative real-time PCR in myocardium of mice

Gene name	Forward sequence 5'→3'	Reverse sequence 5'→3'
PPARα	TCGGCGAACTATTCGGCTG	GCACTTGTGAAAACGGCAGT
PGC1α	CGGAAATCATATCCAACAG	TGAGGACCGCTAGCAAGTTTG
CD36	GTAGTGAGGAATTCAGGGTA	TCTGTTGAGACTCTGAAAGG
ACOX1	GGGAGTGCTACGGGTTACATG	CCGATATCCCCAACAGTGATG
CPT1α	AGAATCTCATTGGCCACCAG	CAGGGTCTCACTCTCCTTGC
Cyp4a10	CCAGGAACTGCATTGGGAAA	GACCCTGGTAGGATCTGGCA
FADS1	ACCCAGCTTTGAACCCACC	GAGGCCCATTCGCTCTACTG
FADS2	GCTCTCAGATCACCGAGGAC	AGTGCCGAAGTACGAGAGGA
Trx1	TGCAGAAAACCTTTGTTCA	TGGAACTGGAGGAACAAGTAGCT
TrxR	CTACAGACCATTGCCTTGCT	ACCTCCTACCCACAAGATCC
GR	TCCGTGCCTGGTAGGAAGCC	GCAGCGATTGCAACTGGGGT
p53	CCCCTGTCATCTTTTGTCCCT	AGCTGGCAGAATAGCTTATTGAG
β-actin	CCTGAGCGCAAGTACTCTGTGT	GCTGATCCACATCTGCTGGAA

Abbreviations: ACOX1: Acyl-CoA Oxidase 1; CD36: Cluster of differentiation 36; CPT1α: Carnitine palmitoyltransferase I α; Cyp4a10: Cytochrome P450, family 2, subfamily a, polypeptide 10; FADS1: Fatty acid desaturase 1; FADS2: Fatty acid desaturase 2; GR: glutathione reductase; PGC1α: Peroxisome proliferator-activated receptor-γ coactivator 1-α; PPARα: Peroxisome proliferator-activated receptor α; Trx1: thioredoxin-1; TrxR: thioredoxin reductase.

**Table 3:** Primers used in Chromatin immunoprecipitation assay

Location	Forward sequence 5'→3'	Reverse sequence 5'→3'
330	TTCCCACCGACTGTTCTC	CTCCTCGATGCCCATTTAGT
470	GATCTAGACCAGCTCAACGA	GTAAACTGAGGCGGGTTAG
910	ATCGCCACAAATAGCATAGT	TTGTGTCGTCCTTGGATCT
1030	GGGTTTAAAAGACGTCCCTG	CACTATGCTATTTGTGGCGA
1180	AGTAGGCAAGTAGGGAATGT	CAGGGACGTCTTTTAAACCC
1520	ATATACAGGCCAGGGTAGGA	GCACAGTGACCTGTCTGTAT
1840	TTCTCATCTTGAAACCTTGGG	TCTGCTGTTAACCCAGTTCT

## Abbreviations

ATP: adenosine triphosphate
ACC: Acetyl-CoA carboxylase
ATCC: American Type Culture Collection
BSA: bovine serum albumin
CPT1α: carnitine palmitoyltransferase 1α
DMEM: Dulbecco's Modified Eagle's Medium
DMSO: dimethyl sulfoxide
E/A ratio: ratio of the early (E) to late (A) ventricular filling velocities
ETC: electron transport chain
EF: ejection fraction
FBS: fetal bovine serum
h: hour
LV: left ventricular
IVRT: isovolumic relaxation time
LVID-d: LV end-diastolic diameter
MEM: Minimum Essential Medium Eagle
MPI: myocardial performance index
OCR: oxygen consumption rates
PBS: phosphate-buffered saline
PPARα: peroxisome proliferator-activated receptor α
PFTα: Pifithrin-α
PGC1α: αproliferator-activated receptor gamma coactivator 1 α
Rap1: repressor activator protein 1
Rap1^+/+^: Rap1 wildtype
Rap1^-/-^: Rap1 knockout
ROS: reactive oxygen species
SA-β-Gal: Senescence associated β-galactosidase
