# Supplementary material for: Deficiency of telomere-associated repressor activator protein 1 precipitates cardiac aging in mice via p53/PPARα signaling
Source: Theranostics. 2021 Mar 4;11(10):4710–27. doi: 10.7150/thno.51739 (PMC7978321; doi:10.7150/thno.51739)
Supplement: Supplementary file 1 — Supplementary figures and table. [file thnov11p4710s1.pdf]

Supplementary material

Deficiency of Telomere-associated Repressor Activator Protein 1 Precipitates Cardiac Aging in Mice *via* p53/PPARα Signalling

Yin Cai, Hao Liu, Erfei Song, Lin Wang, Jindong Xu, Yi He, Dengwen Zhang, Liyan Zhang, Kenneth King-yip Cheng, Leigang Jin, Min Wu, Shiming Liu, Dake Qi, Liangqing Zhang, Gary D. Lopaschuk, Sheng Wang, Aimin Xu, Zhengyuan Xia

Supplemental Table

Table S1 Basic information of recruited patients.

	Young group (n = 7)	Middle age/aged group (n = 5)
Age (year)	31.3 ± 1.7	58.8 ± 3.9*
BW (kg)	55.4 ± 12.7	64.0 ± 8.2
BP (systolic, mmHg)	118 ± 12	140 ± 22
BP (diastolic, mmHg)	72 ± 9	80 ± 11
LVEF%	56.8 ± 7.9	40.5 ± 8.6

Data are shown as means ± S.E.M; *$P < 0.05$ middle age/aged group *vs*. young group. Abbreviations: BW = body weight; BP = blood pressure; LVEF = left ventricular ejection fraction.

Supplemental Figures

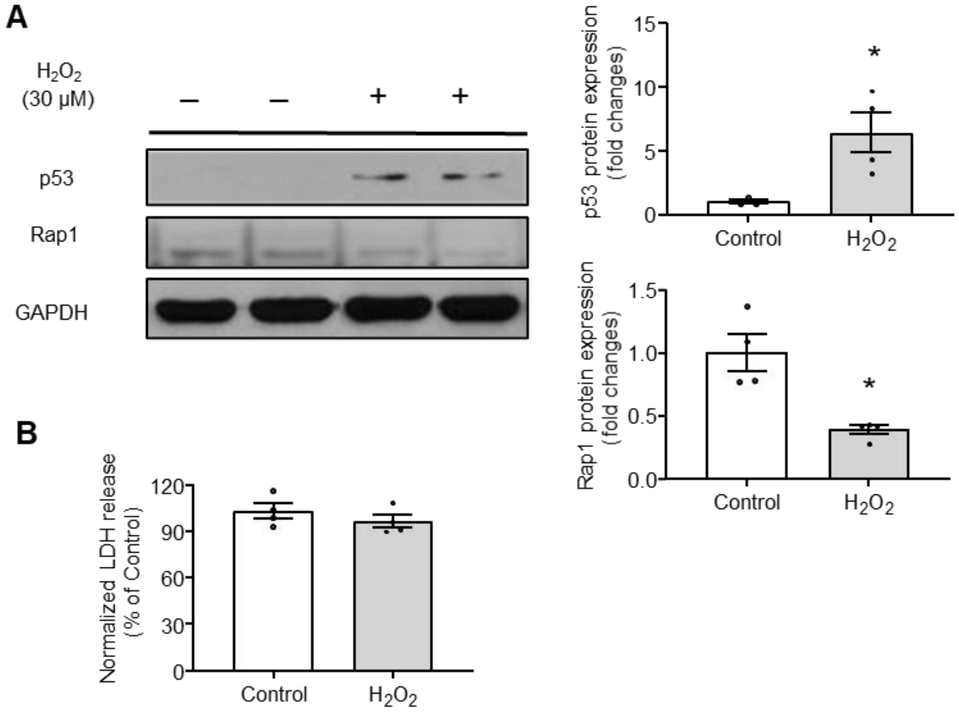

Figure S1. Protein expression of Rap1 was reduced in H_2O_2-treated H9C2 cells. (A) Representative Western blots of p53, Rap1 expression and (B) lactate dehydrogenase (LDH) release in H9C2 cells stimulated with or without H_2O_2 [24 h following 2 h H_2O_2 (30 μM) treatment], n = 4; Protein presence of p53 and Rap1 was normalized to GAPDH. Data are shown as means ± S.E.M; $^{*}P < 0.05$ H_2O_2 *vs*. Control.

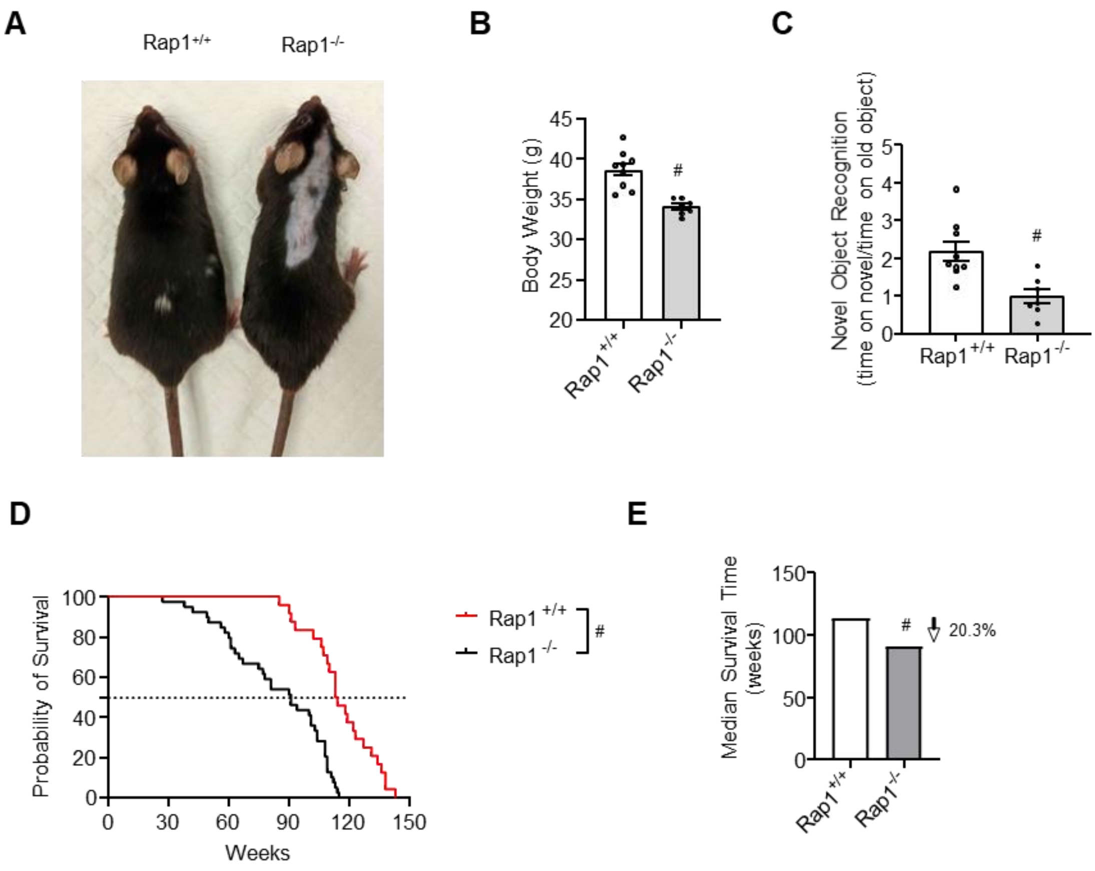

Figure S2. Rap1 deficiency leads to more pronounced age-associated phenotypes. (A) Representative photograph of male Rap1$^{+/+}$ (left) and Rap1$^{-/-}$ (right) mice at 20-months of age. (B) Body weight of Rap1$^{+/+}$ (n = 9) and Rap1$^{-/-}$ (n = 7) mice at 20-months of age. (C) Novel object recognition test (time spends on novel/time spends on old object) in the aged Rap1$^{+/+}$ (n = 9) and Rap1$^{-/-}$ (n = 7) mice (20-months of age). (D) Kaplan–Meyer survival curves and (E) median survival from Kaplan–Meyer plots of male Rap1$^{+/+}$ (n = 24) and Rap1$^{-/-}$ (n = 40) mice. Data are shown as means ± S.E.M.; $^{\#}$P < 0.05 Rap1$^{-/-}$ *vs*. Rap1$^{+/+}$.

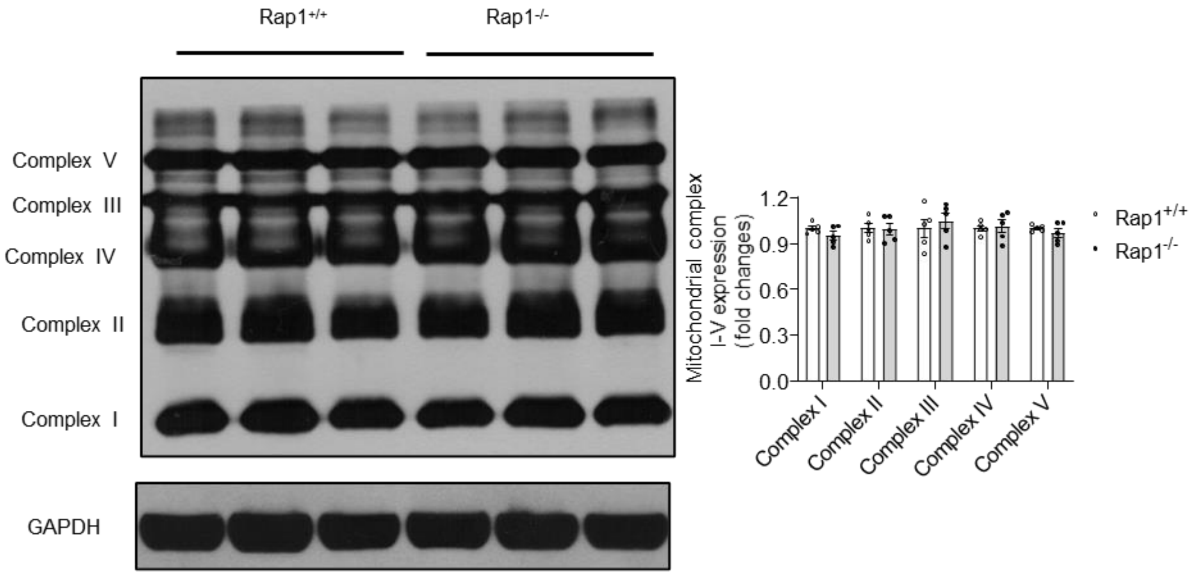

Figure S3. Rap1 does not alter the protein presence of mitochondrial complex I-V. Representative Western blots of mitochondrial complex I-V in the hearts of $Rap1^{+/+}$ and $Rap1^{-/-}$ mice at 12-month of age. Protein presence of mitochondrial complex I-V was normalized to GAPDH. Data are shown as means ± S.E.M. n = 5.

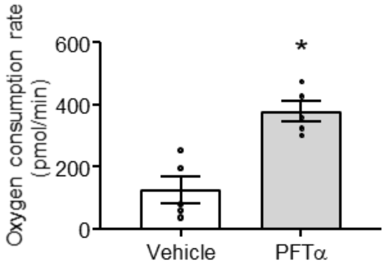

Figure S4. Pharmacological inhibition of p53 enhances mitochondrial respiration in wildtype primary cardiomyocytes. Oxygen consumption rates (OCR) using fatty acids as a substrate were measured under basal conditions in the primary cardiomyocytes from the hearts of $Rap1^{+/+}$ mice at 12-month of age, with or without treatment of PFTα (a selective p53 inhibitor, 10 µM, 12 h). Data are shown as means ± S.E.M. $^{*}P < 0.05$ PFTα *vs*. Vehicle. n = 5.
